# A Novel Method for Practicing Fascial Closure with Suture

**DOI:** 10.7759/cureus.7803

**Published:** 2020-04-23

**Authors:** Tye Patchana, Paras Savla, James Brazdzionis, James Wiginton, Ariel Takayanagi, Bailey Zampella, Michael Schiraldi, Christopher King

**Affiliations:** 1 Neurosurgery, Riverside University Health System Medical Center, Moreno Valley, USA; 2 Neurosurgery, Desert Regional Medical Center, Palm Springs, USA; 3 Neurosurgery, Redlands Community Hospital, Redlands, USA; 4 Neurosurgery, Aurora Medical Center, Summit, USA

**Keywords:** fascia, fascial closure, resident education, neurosurgery, spine surgery

## Abstract

Closure of the fascial layer can be challenging to learn for junior level residents. Wound dehiscence involving the fascial layer can lead to complicated clinical courses for patients, including readmission to the hospital, wound vacuum placement, antibiotic regimens, and re-operation. Typical suturing techniques taught in medical school focus more on basic techniques of suture placement such as interrupted or running techniques. The aim of this study is to introduce a method of practicing fascial closure using easily obtainable items. Though there is no substitute for placement of suture and closure of fascia in vivo, this method allows one to practice the motor repetition of fascial suture placement and provides one with the ability to check their work.

## Introduction

Closure of the dorsal spinal fascial layer can be challenging to learn for junior level residents. Typical suturing techniques taught in medical school focus more on basic techniques of suture placement such as interrupted or running techniques. The aim of this study is to introduce a method of practicing fascial closure using easily obtainable items. Though there is no substitute for placement of suture and closure of fascia in vivo, this method allows one to practice the motor repetition of fascial suture placement and provides one with the ability to check their work.

Fascia, derived from Latin for “band,” is a connective tissue responsible for the compartmentalization of anatomical structures within the body [1]. Primarily composed of collagen, it lends structural support and flexibility to the structures found deep to the skin. Density of the fascial layer is highest in the lumbar region and has been found to be related proportionally to the amount of physical activity [2]. The thoracolumbar fascia further separates the superficial and deep muscles of the back. Proper exposure during spinal procedures is predicated on the disruption of the thoracolumbar fascia.

The importance of proper closure and maintenance of the fascial plane following surgical procedures cannot be over-emphasized. Wound dehiscence involving the fascial layer can lead to complicated clinical courses for patients, including, but not limited to, readmission to the hospital, wound vacuum placement, antibiotic regimens, and re-operation [3]. Those at risk of facial dehiscence include obese and immunocompromised patients, which make up an increasing population undergoing spinal procedures. Though there are methods of prevention of dehiscence, such as mesh placement, prevention of dehiscence begins with proper closure, which may be obtained by appropriate approximation and distribution of tension. Previously, placement of synthetic mesh within the thoracolumbar fascia has been investigated as a method of reducing pain, improving cosmetic results, and improving clinical outcomes [4].

The method described in this study was born out of a need for the authors to practice fascial closure and has been employed at our residency program’s weekly skills practice for junior residents. This method can also be readily available in resource-limited countries. A perusal on various medical specialty, as well as commercial websites, demonstrates an array of different systems being sold to practice such skills, most of which can be costly. We aim to provide a simple, cheap, and useful way to practice fascial closure that can translate to improved technique in the operating room (OR).

## Materials and methods

**Figure 1:**
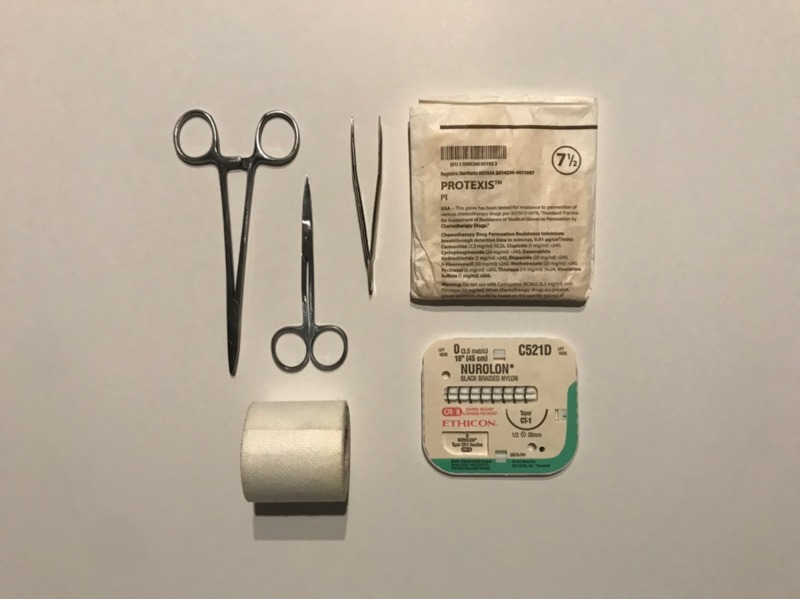
General supplies needed for a fascial closure setup: a needle drive, scissors, forceps, operating room gloves, sutures, and tape.

3M Medipore Soft Cloth Surgical tape (3M, St. Paul, MN, USA) was used. Though a variety of tapes may be used, this particular version has several features that make it useful for practicing fascial closure. It has diagonal and cross-stretching capabilities, which make it similar to biological structures. It can be folded to decrease stretch depending on the rigidity required and the type of suturing technique being practiced. Additionally, it is hypo-allergenic and readily attainable at most hospitals. It also has perforations, which make it easy to tear to create planes of desired sizes. We employed instruments from a standard laceration tray, including needle drivers, scissors, forceps, and standard OR gloves, as well as braided suture and 3M Medipore tape (Figure 1). In our demonstration, 0 Nurolon suture (Ethicon, Somerville, NJ, USA) was used, as it was readily available at our institution secondary to expiration.

## Results

For demonstration purposes, a small cardboard box was used as a base upon which the tape to be approximated by suture was affixed. A standard textbook has also been used by the authors, though any platform to provide a weighted base can be used. It is helpful to secure the base using additional tape (not shown in our demonstration) in order to create a fixed surface to practice edge approximation. This can be placed on a table or the operator’s lap, or can be manipulated into a variety of different orientations to simulate the operator’s positioning in the OR.

**Figure 2:**
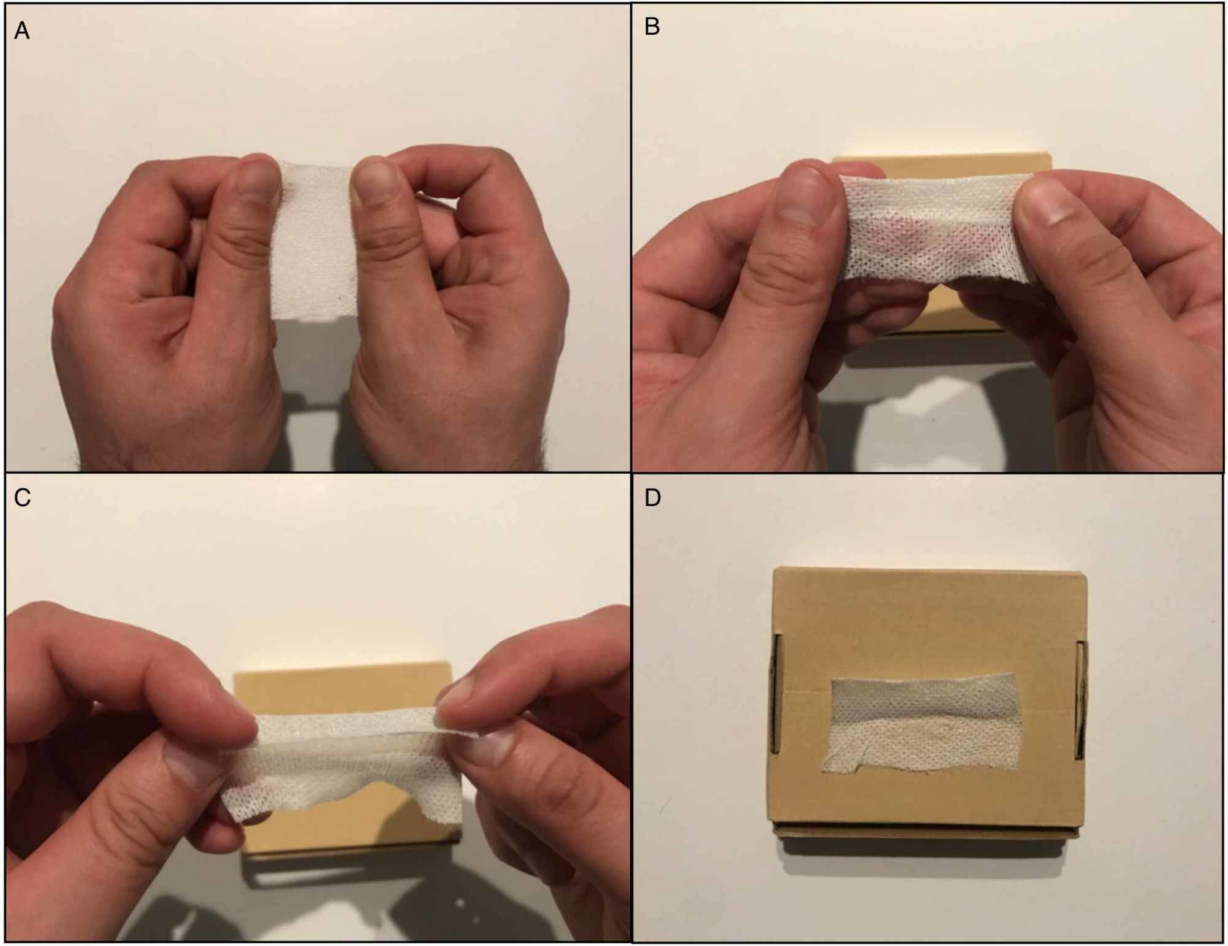
Producing two edges to suture together. (A) Cross-stretching ability of the tape. (B) One end of the tape, approximately one-third, is folded over itself. (C) Additional folds can be added for increased rigidity. (D) Demonstrates placement of tape onto an object. In this case, a cardboard box was used.

**Figure 3:**
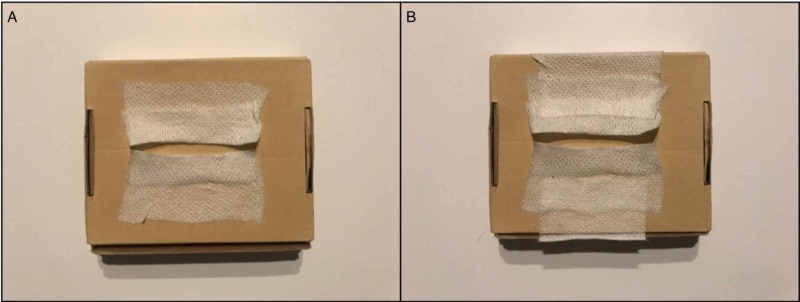
Tape applied to the base. In this case, a cardboard box was used. (A) Application of the tape to the cardboard box. (B) Additional tape has been used to secure each piece for optimal stretch. Space between the two folded ends of tape can be adjusted to make a more challenging approximation between the ends.

In order to simulate closure, the following technique was employed. Upon identification of the chosen base structure, the cloth tape was torn in two symmetric pieces to the desired length. The one-third end of the tape was then folded over itself and fixed to the box in parallel with the space between the tape as the desired distance of closure. From there, the two pieces of tape were sutured together with tension applied to ensure appropriate locking and maintenance of approximation of the surrogate tissue (Figures 2, 3).

**Figure 4:**
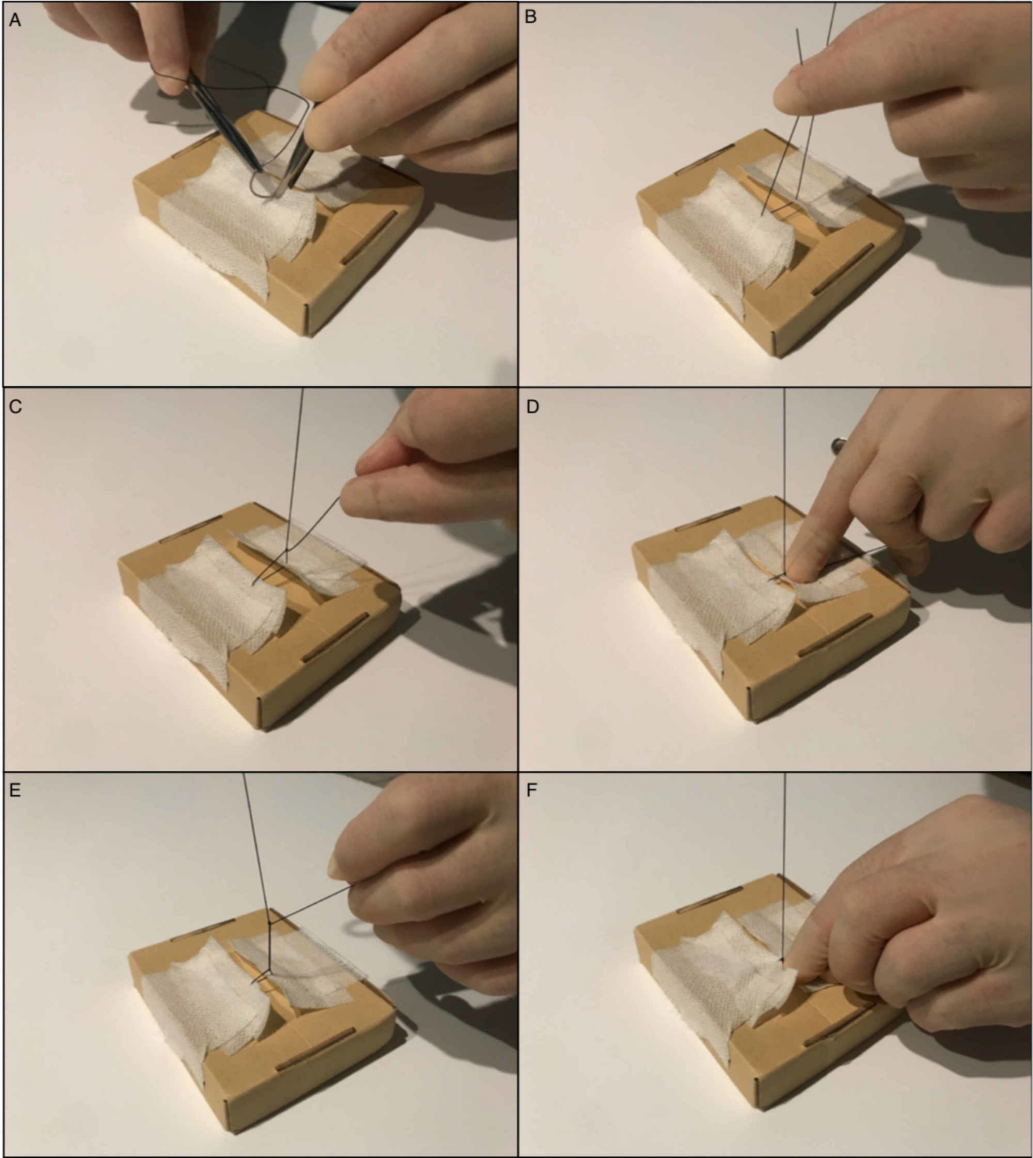
Standard fascial closure technique is used to approximate the edges. (A-F) Standard tying of knot, using upward tension with the non-tying hand as the knot is cinched down.

**Figure 5:**
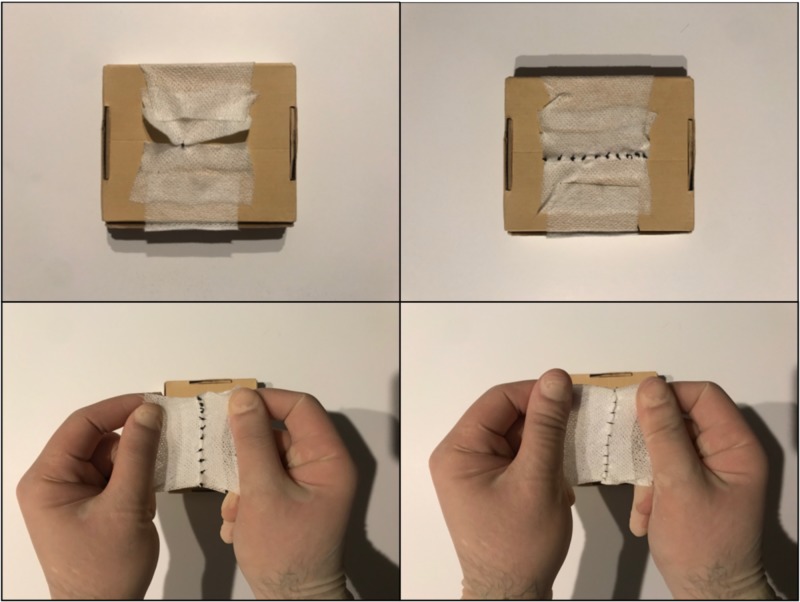
Once the edges are approximated, the tape may be removed to view the underside. This demonstrates ability to check one's work and to inspect integrity of the closure with added tension.

The standard fascial closure technique was employed, involving one throw of suture through both simulated wound edges. One-handed surgeon's knot was practiced in our demonstration. The first knot was thrown, and pressed down with the non-tying hand. A second knot was then thrown and simultaneously pushed down with the non-tying hand while the tying hand cinched the remaining suture with upward tension. This second knot will lock the suture in place. Additional knots in the opposite directions may then be thrown for added mechanical strength. This is repeated until complete approximation is achieved. After placing the desired number of sutures, the tape may be removed and pulled to identify the strength of closure (Figures 4, 5).

## Discussion

The aim of this study is to make practicing closure of the fascia readily accessible, cost-effective, and simple, using just a few items that can be obtained at almost any hospital. Several methods exist for practicing the suturing technique, including several commercially available silicone-based products, practicing on pig's feet, and cadaveric laboratories. There are advantages and limitations to each of these methods. Previously, practice boards and suture practice cards were employed in general surgery and microsurgical training [5,6]. Similar to these, our setup has the advantage of not being compromised by repetitive use as well as being non-perishable compared to practice on animal tissues. Of course, there is no substitute for practicing the suturing technique in vivo within the OR. Though our method is limited in that regard, we believe that the principles of fascial closure can be practiced in an economical and readily available way.

We believe that practicing approximating the edges of tape can help prepare residents for the OR, where there must be certainty in the integrity of surgical knots. Additionally, this method offers versatility in practicing specific skills. One may place more distance between the edges of tape and practice retention suture for gross approximation followed by standard closure.

The importance of this skill cannot be over-emphasized. In the Neurosurgery Residency Program at our institution, Riverside University Health System, we have instituted the aforementioned method at our weekly “Skills Day” meetings, with junior residents being taught by attending neurosurgeons and senior neurosurgery residents.

## Conclusions

The aim of our study was to introduce a method of practicing the important skill of facial closure using readily obtainable items. Closure of this layer can be challenging to learn for junior level residents. Typical suturing techniques taught in medical school focus more on basic techniques of suture placement such as interrupted or running techniques. Though there is no substitute for the placement of suture and closure of fascia in vivo, this method allows one to practice the motor repetition of fascial suture placement and provides one with the ability to check their work. It has the advantage of being readily obtainable, non-perishable, and economical.

## References

[1] Gilroy AM, MacPherson BR, Schuenke M, Schulte E, Schumacher U (2016). Atlas of Anatomy. 3rd Edition.

[2] Schleip R, Klinger W, Lehmann-Horn F (2005). Fascia is able to contract in a smooth muscle-like manner and thereby influence musculoskeletal mechanics. Med Hypotheses.

[3] Feng C, Qianqian S, Jianhua H, Yu Z, Yipeng W, Jianguo Z, Guixing Q (2019). Treatment experience for full-thickness wound dehiscence with cerebrospinal fluid leakage following posterior primary spine surgery. Medicine (Baltimore).

[4] Jentzsch T, Geiger J, and Werner CML (2016). Synthetic meshes in the treatment of postoperative fascial dehiscence of the spine [online ahead of print]. J Back Musculoskelet Rehabil.

[5] Usón J, Calles MC (2002). Design of a new suture practice card for microsurgical training. Microneurosurgery.

[6] Weeks D, Kasdan ML, Wilhelmi BJ (2020). Design of a new suture practice card for microsurgical training. Eplasty.

